# Evolution of facial muscle anatomy in dogs

**DOI:** 10.1073/pnas.1820653116

**Published:** 2019-06-17

**Authors:** Juliane Kaminski, Bridget M. Waller, Rui Diogo, Adam Hartstone-Rose, Anne M. Burrows

**Affiliations:** ^a^Centre for Comparative and Evolutionary Psychology, Department of Psychology, University of Portsmouth, Portsmouth P01 2DY, United Kingdom;; ^b^Department of Anatomy, Howard University College of Medicine, Washington, DC 20059;; ^c^Department of Biological Sciences, North Carolina State University, Raleigh, NC 27695;; ^d^Department of Physical Therapy, Duquesne University, Pittsburgh, PA 15228

**Keywords:** facial muscle anatomy, domestic dogs, wolves, domestication

## Abstract

Domestication shaped wolves into dogs and transformed both their behavior and their anatomy. Here we show that, in only 33,000 y, domestication transformed the facial muscle anatomy of dogs specifically for facial communication with humans. Based on dissections of dog and wolf heads, we show that the levator anguli oculi medialis, a muscle responsible for raising the inner eyebrow intensely, is uniformly present in dogs but not in wolves. Behavioral data, collected from dogs and wolves, show that dogs produce the eyebrow movement significantly more often and with higher intensity than wolves do, with highest-intensity movements produced exclusively by dogs. Interestingly, this movement increases paedomorphism and resembles an expression that humans produce when sad, so its production in dogs may trigger a nurturing response in humans. We hypothesize that dogs with expressive eyebrows had a selection advantage and that “puppy dog eyes” are the result of selection based on humans’ preferences.

## Significance

Dogs were shaped during the course of domestication both in their behavior and in their anatomical features. Here we show that domestication transformed the facial muscle anatomy of dogs specifically for facial communication with humans. A muscle responsible for raising the inner eyebrow intensely is uniformly present in dogs but not in wolves. Behavioral data show that dogs also produce the eyebrow movement significantly more often and with higher intensity than wolves do, with highest-intensity movements produced exclusively by dogs. Interestingly, this movement increases paedomorphism and resembles an expression humans produce when sad, so its production in dogs may trigger a nurturing response. We hypothesize that dogs’ expressive eyebrows are the result of selection based on humans’ preferences.

The dog−human bond is unique and diagnostic of the evolution of human cultures. Dogs were domesticated over 33,000 y ago (1), and, during that time, selection processes have shaped both their anatomy and behavior and turned them into human’s best friend (2). The most remarkable among dogs’ behavioral adaptations, as a result of selection during domestication, is their ability to read and use human communication in ways that other animals cannot (3, 4). Dogs are more skillful in using human communicative cues, like pointing gestures or gaze direction, even than human’s closest living relative, chimpanzees, and also than their own closest living relatives, wolves, or other domesticated species (5). Recent research suggests that eye contact between humans and dogs is crucial for dog−human social interaction. Dogs, but not wolves, establish eye contact with humans when they cannot solve a problem on their own (6, 7). Eye contact also helps dogs to know when communication is relevant and directed at them, as dogs tend to ignore human pointing gestures when the human’s eyes are not visible (8, 9). Dogs, but not wolves, seem to be motivated to establish eye contact with humans from an early age (10, 11), and dogs’ motivation to establish eye contact with humans seems to be an indicator of the level of attachment between humans and dogs (12). Thus, mutual gaze between dogs and humans seems to be a hallmark of the unique relationship between both species during human cultural evolution.

Nagasawa et al. (13) showed that, between dogs and humans (but not wolves and humans), mutual gaze seems to lead to an oxytocin feedback loop analogous to the one that exists between human mothers and infants. Oxytocin has a fundamental role during affiliative behaviors in mammals and during the onset of maternal behavior and mother−infant attachment (14). Similarly, mutual gaze between dogs and humans seems to trigger an increase of oxytocin in both species, which then increases the motivation to establish eye contact (13). As this cross-species oxytocin loop can be found in dogs and humans, but not between dogs’ closest living relative (the wolf) and humans, selection processes during domestication must have played an important role whereby dogs hijacked the human caregiving response (15). The most likely evolutionary scenario is that dogs’ ancestor must have, to some extent, expressed characteristics that elicited a caregiving response from humans. Humans then consciously or unconsciously favored and therefore selected for those characteristics, leading to the analogous adaptations we see in dogs today.

Selection for traits that facilitate eye contact between dogs and humans might have, therefore, led to 1) anatomical differences in the facial musculature around the eyes between dogs and wolves and 2) behavioral differences between the species in terms of how they use these muscles to promote eye contact. We know that humans favor dogs that show paedomorphic (infant-like) anatomical features like a large forehead, large eyes, and so on; in studies asking people to select pictures presenting dog (or cat) faces, people prefer the faces that present paedomorphic features over others (16). Importantly, paedomorphic facial features can be even further exaggerated by facial muscle movements, which act to enhance the appearance of specific facial features (particularly the eyes). Waller et al. (17) showed that a specific facial muscle movement around the eyes (which they termed AU101: inner eyebrow raise) seems to be particularly attractive to humans. The movement makes the eyes appear bigger, hence more infant-like and potentially more appealing to humans. This inner brow raise also resembles a facial movement humans produce when they are sad, potentially eliciting a nurturing response from humans (17, 18). The study showed that dogs that produce this facial movement more were rehomed from a shelter more quickly than those that produced the movement less often, suggesting that the production of this eye movement gives dogs a potential selection advantage. No other facial movement had the same effect (17). However, thus far, it has been unknown whether domestication has shaped this phenomenon, and whether dogs show marked differences from wolves in anatomy and behavior in relation to this facial movement.

## Results

To determine whether domestication has shaped facial muscles to facilitate dog−human communication in this way, we 1) conducted a detailed comparative facial dissections of gray wolves (*Canis lupus*, *n* = 4) and domestic dogs (*Canis familiaris*, *n* = 6) and 2) quantified wolves’ and dogs’ AU101 facial movements in their frequency and intensity during social interactions with humans in both wolves (*C. lupus*, *n* = 9) and domestic dogs (*C. familiaris*, *n* = 27).

**Fig. 1.**
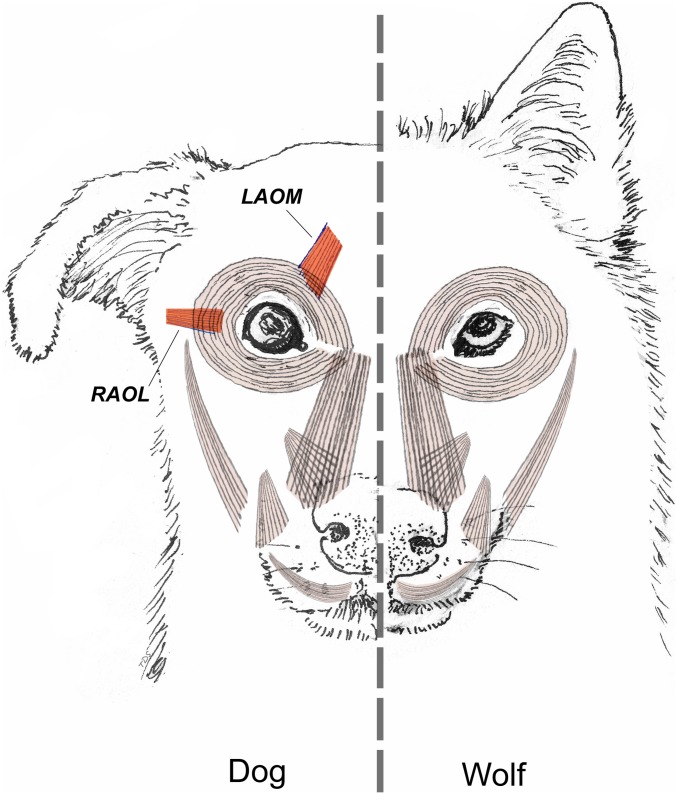
Facial musculature in the wolf (*C. lupus*) (animal’s left) and dog (*C. familiaris*) (right) with differences in anatomy highlighted in red. Image courtesy of Tim D. Smith (Cambridge University Press, Cambridge, UK).

**Fig. 2.**
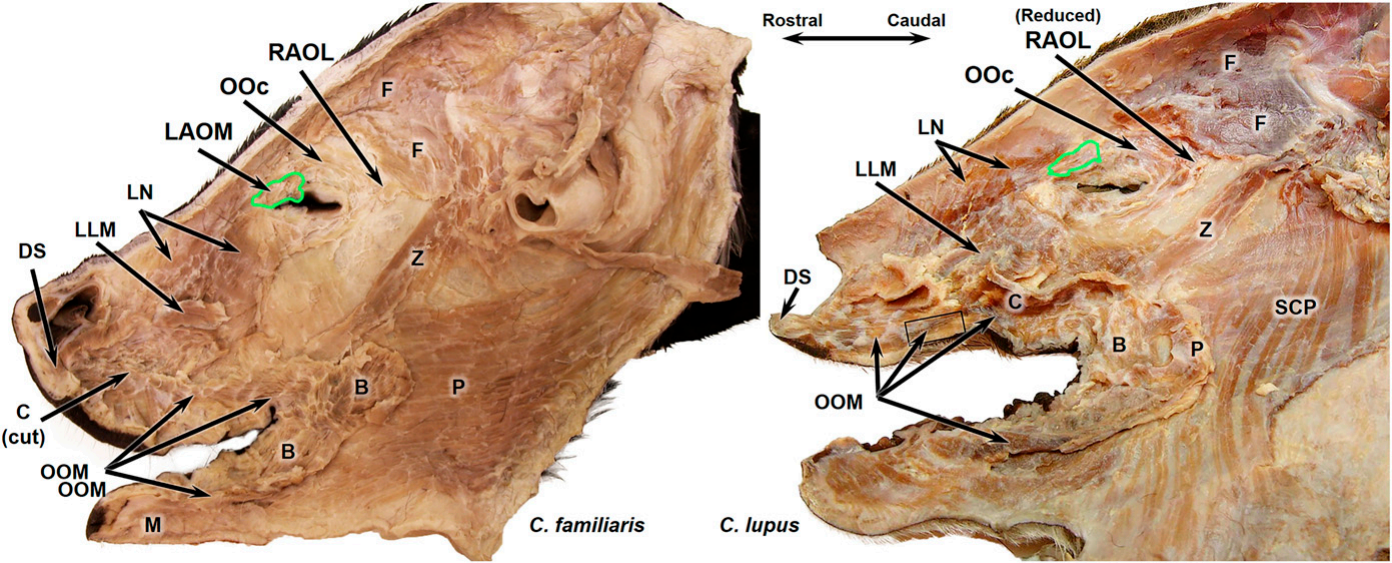
Right-side facial masks from domestic dog (*C. familiaris*) and gray wolf (*C. lupus*). B, buccinator muscle; C, caninus muscle; DS, depressor septi muscle; F, frontalis muscle; LLM, levator labii maxillaris (deep to LN); LN, levator nasolabialis muscle; M, mentalis muscle; OOc, orbicularis oculi muscle; OOM, orbicularis oris muscle; P, platysma muscle (note that this muscle is cut away in the gray wolf to reveal the SCP); SCP, sphincter coli profundus muscle; Z, zygomaticus muscle. Green line encircles the LAOM in the domestic dog and the typically reduced LAOM in the gray wolf. Terminology based on ref. 33.

The main finding is that facial musculature between domestic dogs and gray wolves was relatively uniform and differed only around the eye (Fig. 1 and Table 1). While the levator anguli oculi medialis muscle (LAOM) was routinely present in dogs, in the gray wolves, it was typically represented only by scant muscle fibers surrounded by a high quantity of connective tissue. In the wolves, a tendon was sometimes observed that blended with the medial aspect of the fibers of the orbicularis occuli muscle, near the region where an LAOM would normally be expected (Fig. 2). Thus, wolves have less ability to raise the inner corner of their brows independent of eye squinting relaxation—the anatomical basis for the difference in expression of the AU101 movement.

**Table 1.** Muscle presence/absence between gray wolf (*C. lupus*) and domestic dog (*C. familiaris*)

Muscle	*C. lupus* (*n* = 4)	*C. familiaris* (*n* = 6)
Zygomaticus	P	P
Orbicularis occuli	P	P
RAOL	V*	P
LAOM	A^†^	V^‡^

“P” indicates that the muscle is always present; “V” indicates that the muscle is variably present; “A” indicates that this muscle is mostly present (see ref. 2).

* This muscle was absent in one of the wolf specimens.

^†^ This muscle was never present in the gray wolf as a separate muscle but instead appeared as a small tendon incompletely separated from the orbicularis oculi muscle.

^‡^ This muscle was consistently present as an independent muscle in all specimens except for one, a Siberian husky, where it could not be located.

Other facial muscles around the eye, for instance, the orbicularis oculi muscle and frontalis muscle, that did not differ either within or between species. The only exception was the retractor anguli oculi lateralis muscle (RAOL). RAOL was highly variable in size and presence (Table 1)—present in most of the gray wolves but typically more gracile than in the domestic dog, consisting of scant bundles of muscle fibers. The RAOL pulls the lateral corner of the eyelids toward the ears. All domestic dogs routinely possessed this muscle, except for the Siberian husky specimen, which interestingly belongs to the more ancient dog breeds, more closely related to wolves than many other breeds (19). Thus, most of the dogs in our sample had a greater ability than gray wolves to pull the lateral corners of their eyelids posterolaterally toward their ears. There was no other substantial variability in the facial musculature within the gray wolf sample, except for the RAOL, which was present in only three of the four specimens.

These anatomical differences between dogs and wolves correspond to our behavioral analysis of the facial movements oriented toward a human in 27 dogs (*C. familiaris*) and nine wolves (*C. lupus*). The dogs came from several shelters across the United Kingdom and were observed by a stranger who approached their kennel and filmed their behavior for 2 min each. The wolves came from two different wolf parks where they lived in groups and were filmed by a stranger individually for ∼2 min each. We analyzed the frequencies of AU101 movements both species produced as well as the level of intensity of those movements, from low intensity (A) to high intensity (E). We first compared the frequency of AU101 between species and found that, overall, dogs produced significantly more AU101 movements [median (Mdn) = 10] than wolves (Mdn = 2, Mann−Whitney: *U* = 36, z = −3.13, *P* = 0.001). We then looked at the frequencies of AU101 movements by intensity level (A to E). Comparisons revealed that, while dogs and wolves seem to produce movements at lowest intensity (A) at the same frequency (Mdn dogs = 0.167, Mdn wolves = 0.75, Mann−Whitney: *U* = 74.5, z = −1.74, *P* = 0.086), all higher intensity levels are produced at higher frequency in dogs (intensity level B: Mdn dogs = 0.32, Mdn wolves = 0, Mann−Whitney: *U* = 67.5, z = −1.99, *P* = 0.047; intensity level C: Mdn dogs = 0.17, Mdn wolves *=* 0, Mann−Whitney: *U* = 32.5, z = −3.35, *P* = 0.001) or produced exclusively by dogs (intensity levels D and E).

## Discussion

Overall, our findings therefore show that selection pressures during domestication have shaped the facial muscle anatomy of dogs. While we have known for a long time that dog body shape and skeletal anatomy has been subject to artificial selection pressures, this is evidence that anatomical differences are also seen in the soft tissue—a striking difference for species separated only about 33,000 y ago. Soft tissue changes are inherently hard to document given that soft tissues do not readily fossilize. Moreover, we show that these remarkably fast muscular changes can be linked directly to enhanced social interaction with humans. The rest of the facial anatomy did not differ between the species, so this anatomical difference translates to behavioral differences between dogs and wolves as dogs produce more common and exaggerated AU101 eyebrow facial movements than do wolves. Differences in intensity levels could also be due, in part, to a differential presence of connective tissue in the face between dogs and wolves, which might explain why, at very low intensity, no differences can be found between both species.

The AU101 movement causes the eyes of the dogs to appear larger, giving the face a more paedomorphic, infant-like appearance, and also resembles a movement that humans produce when they are sad (20). It therefore has the potential to elicit a caregiving response from humans, giving individuals that inherit the trait a selection advantage with humans. The likely evolutionary scenario was that humans consciously or unconsciously preferred (and therefore cared more for) individuals that produced the movement, which led to a selection advantage and manifestation of the trait. Since Waller et al. (17) found that dogs that produce this facial movement more were rehomed from a shelter more quickly, if that rehoming provides a genetic survivability advantage (i.e., if rehomed dogs are not sterilized and are more likely to produce offspring), then this type of selection is still happening to some extent.

There might be an additional reason why the AU101 movement is potentially of great significance for the dog−human bond: not just because it might elicit a caring response, but also because it might play a role during dog−human communicative interactions. In humans, eyebrow movements are seen as part of a set of cues, so-called ostensive cues, which are of particular significance during communicative interactions (21). In humans, eyebrow movements seem to be particularly relevant to boost the perceived prominence of words and act as focus markers in speech (22, 23). During communicative interactions, observers seem to pay particular attention to the upper facial area for prominence detection (23), and humans prefer utterances in which pitch and eyebrow movements are aligned on the same word and downscale the prominence of unaccented words in the immediate context of the eyebrow-accented words (24). Ostensive cues, like eyebrow movements, are seen as particularly relevant in the so-called pedagogical context, that is, when infants are learning something from others like, for instance, the meaning of words (25). The hypothesis is that humans are specifically adapted to being attentive to these kinds of ostensive cues and that this is a uniquely human feature (21).

Thus, it could be that humans consciously or unconsciously selected for exaggerated eyebrow movements in dogs, as they would be perceived as markers during communicative interactions. During communicative interactions, human observers not only pay particular attention to the upper facial area of other humans but also automatically pay attention to the upper facial area, in particular the eye region, while looking at pictures of animals, including dogs (26). As dogs seem to be specifically selected to respond to (and attend to) communicative interactions with humans, flexible eyebrow movements in dogs could have been a side product of that selection process.

Wolves, in comparison with other canids, are described as having an intense gaze-signaling face (27). Wolves have a lighter-colored iris compared with other canid species, which, as shown by Ueda et al. (27), correlates with longer-duration facial gaze signals. While this might have formed a basis for human attention to the wolf eyes, selection for more exaggerated eyebrow movements could have been what created the illusion of human-like communication. The heightened eyebrow movements may have been perceived by humans as markers similar to those established during human−human communicative interactions. Interestingly a recent study shows that dogs seem to produce significantly more AU101 when a human is looking at them, which might support the hypothesis that this is the context within which this trait evolved (28).

An alternative hypothesis could be that more-exaggerated AU101 movements are attractive for humans because they expose the white parts of the sclera in the dogs’ eyes. Humans, unlike other primates which have gaze-camouflaging eyes, have a visible white sclera (29, 30). The depigmentation and visibility of the human sclera is hypothesized to be an adaptation to support cooperative social and communicative interactions (“cooperative eye hypothesis”) as it helps indicate gaze direction much more saliently (30, 31). Indeed there is evidence that humans have a preference for interacting with targets with a visible white sclera (29). When presenting participants with a series of stuffed animals (e.g., dogs), which only varied around the eyes in eye size, color, and the presence of a white sclera, Segal et al. (29) showed that children and adults significantly preferred animals with a visibly white sclera over other targets.

Overall, the data suggest that selection—perhaps mainly unconscious—during social interactions can create selection pressures on the facial muscle anatomy in dogs strong enough for additional muscles to evolve. This opens up interesting questions for future research, such as questions on other domestic species like cats and domestic horses and also breed differences in dogs as, well as questions on the kind of selection pressure necessary for this to emerge. One highly relevant question in this regard would be whether selection for tameness alone might create the same scenario. Here the domesticated silver foxes (32) would be relevant and interesting model taxa.

## Materials and Methods

### Anatomical Data.

The specimens for the comparative facial dissections came from four wild wolves (*C. lupus*) and six domestic dogs (*C. familiaris*; see Table 2 for details on subspecies/breed for each specimen). Two specimens for the wolves were purchased from the taxidermy industry but were not killed for the purpose of this study, and the two other wolf specimens were obtained from the Michigan Department of Natural Resources. Specimens for the dogs were obtained from the National Museum of Health and Medicine (NMHM). All anatomical samples were procured from cadaveric specimens that were not euthanized for our research and were therefore exempt from Institutional Animal Care and Use Committee oversight. The behavioral study was carried out in strict accordance with the recommendations in the Association for the Study of Animal Behaviour/Animal Behaviour Society guidelines for the use of animals in research and was approved by the University of Portsmouth Animal Ethics Committee.

**Table 2.** Subspecies/breed of specimen used in the dissection portion of this study

Specimen	Group	Subspecies/breed	Obtained through
1	*C. lupus*	Alaska Population (free ranging)	Taxidermy Industry
2	*C. lupus*	Alaska Population (free ranging)	Taxidermy Industry
3	*C. lupus*	Michigan Population (free ranging)	Michigan Department of Natural Resources
4	*C. lupus*	Michigan Population (free ranging)	Michigan Department of Natural Resources
5	*C. familiaris*	Labrador Retriever	NMHM
6	*C. familiaris*	Bloodhound	NMHM
7	*C. familiaris*	Chihuahua	NMHM
8	*C. familiaris*	German Shepherd	NMHM
9	*C. familiaris*	Siberian Husky	NMHM
10	*C. familiaris*	Mongrel	NMHM

The main finding is that facial musculature between domestic dogs and gray wolves differed only around the eye. While the LAOM was present in dogs, in the gray wolves, it was never present. In wolves, a tendon was sometimes observed that blended with the medial aspect of the fibers of the orbicularis occuli muscle, near the region where an LAOM would normally be expected (see Fig. 2 for examples of pictures of the dissections).

### Behavioral Data.

Behavioral data were collected from nine wolves from two different animal parks (New Forest Wildlife Park, United Kingdom, for *C.l. occidentalis* and Tierpark Petersberg, Germany, for *C.l. arctos*) and 27 dogs from multiple shelters across the United Kingdom. The dogs were randomly selected from shelters, but formed a rather homogenous group of mainly Staffordshire Bullterriers (*n* = 20) and some mixed-breed dogs (*n* = 7). Each subject was videotaped for 2 min, by a stranger standing in front of the animal at a distance of ∼2 m to 5 m with her body oriented toward the animal. The person filming the dogs was the same for all dogs and was also the person filming the wolves at New Forest Wildlife Park, while the wolves at Tierpark Petersberg were filmed by a different experimenter. The frequency of AU101 movements was coded from videotape by a trained FACS (Facial Action Coding System) coder who was blind to the research hypothesis using Dog FACS [Waller et al. (17), www.animalfacs.com). AU101 movements were coded by intensity ranging from low (A) to high (E) intensity (see Movies S1–S8 for examples). Reliability coding on the intensities of the AU101 movements for the different species was performed by another trained FACS coder who was also blind to the hypothesis of the study. A good degree of reliability was found between measurements. The average measure intraclass correlation coefficient was = 0.76 with a 95% confidence interval.

## Supplementary Material

Supplementary File

Supplementary File

Supplementary File

Supplementary File

Supplementary File

Supplementary File

Supplementary File

Supplementary File

Supplementary File

Supplementary File
