# Supplementary material for: Evolution of facial muscle anatomy in dogs
Source: Proc Natl Acad Sci U S A. 2019 Jun 17;116(29):14677–81. doi: 10.1073/pnas.1820653116 (PMC6642381; doi:10.1073/pnas.1820653116)
Supplement: Supplementary File [file pnas.1820653116.sapp.pdf]

PNAS
www.pnas.org

Supplementary Information for

The evolution of puppy dog eyes

Juliane Kaminski[1]*, Bridget M. Waller[1], Rui Diogo[2], Adam Hartstone-Rose[3] & Anne M. Burrows[4]

Juliane Kaminski
Email: juliane.kaminski@port.ac.uk

This PDF file includes:

Captions for movies S1 to S8
Dataset S1

Other supplementary materials for this manuscript include the following:

Movies S1 to S8

Supplementary Information Text

www.pnas.org/cgi/doi/10.1073/pnas.1820653116

Movie S1. AU 101 movement, Intensity A, Wolf

Movie S2. AU 101 movement, Intensity B, Wolf

Movie S3. AU 101 movement, Intensity C, Wolf

Movie S4. AU 101 movement, Intensity A, Dog

Movie S5. AU 101 movement, Intensity B, Wolf

Movie S6. AU 101 movement, Intensity C, Wolf

Movie S7. AU 101 movement, Intensity D, Wolf

Movie S8. AU 101 movement, Intensity E, Wolf
